# Comparison of the adverse events of anterior cervical disc replacement versus anterior cervical discectomy and fusion: A protocol for a systematic review and meta-analysis of prospective randomized controlled trials

**DOI:** 10.1097/MD.0000000000010015

**Published:** 2018-04-20

**Authors:** He Zhao, Li-Jun Duan, Yu-Shan Gao, Yong-Dong Yang, Xiang-Sheng Tang, Ding-Yan Zhao, Yang Xiong, Zhen-Guo Hu, Chuan-Hong Li, Si-Xue Chen, Tao Liu, Xing Yu

**Affiliations:** a Beijing University of Chinese Medicine, Dongzhimen Hospital; b Department of Orthopedics III, Dongzhimen Hospital Affiliated to Beijing University of Chinese Medicine, Beijing; c Department of Orthopedics, Bayannaoer City Hospital, Bayannaoer City; d School of Basic Medical Sciences, Beijing University of Chinese Medicine; e Department of Orthopedics, China-Japan Friendship Hospital Affiliated to Beijing University of Chinese Medicine, Beijing, China.

**Keywords:** anterior cervical disc replacement, anterior cervical discectomy and fusion, protocol

## Abstract

**Background:**

In the current surgical therapeutic regimen for symptomatic cervical degenerative disc disease, both anterior cervical discectomy and fusion (ACDF) and anterior cervical disc replacement (ACDR) are still widely accepted. However, many complications exist in both surgeries. Therefore, this study aims to compare the adverse events between ACDR and ACDF, and provide vital evidence-based guidance for spine surgeons and designers to evaluation of prognosis and improvement of dynamic devices.

**Methods:**

A systematic review and meta-analysis that will be performed according to the PRISMA. The electric database of PubMed, Medline, Embase, Google Scholar, and Cochrane library will be systematic search. A standard data form will be used to extract the data of included studies. We will assess the studies according to the Cochrane Handbook for Systematic Reviews of Interventions, and perform analysis in RevMan 5.3 software. Fixed effects models will be used for homogeneity data, while random-effects will be used for heterogeneity data. The overall effect sizes will be determined as weighted mean difference (WMD) for continuous outcomes and relative risk (RR) for dichotomous outcomes.

**Result:**

The results of this study will be disseminated via international or national conferences, or submit to peer-reviewed journal in spinal field.

**Conclusion:**

The conclusion of this study will provide key evidence-based guidance for spine surgeons and designers to the evaluation of prognosis and improvement of dynamic devices.

## Abstract 2

Supplemental Digital Content is available in the text

## 1 Introduction

There is no doubt that anterior cervical discectomy and fusion (ACDF), a fusion technique, remains a “golden standard” surgical treatment for symptomatic cervical spondylosis over the past century. The merit is that ACDF is adept at achieving sufficient decompression by raising the height of disc space as well as rebuilding stabilization at an early stage.^[1–3]^ However, with long-term follow-up, ACDF is criticized over changing natural biomechanical environment inducing hypermobility and heightened intradiscal pressures at adjacent levels. These changes in stress and motion profiles are hypothesized to be a primary cause of adjacent-segment degeneration disease (ASDis).^[4–6]^ Anterior cervical disc replacement (ACDR), a nonfusion technique, best known for its motion preserving proprieties and saving overall cervical spine biomechanics may avoid exacerbating adjacent segment degeneration (ASD) and related symptoms.^[7–9]^ At birth, all deem it versatility and are enthusiastic about treating cervical spondylosis with ACDR. However, long-term follow-up disenchants an image that ACDR could replace ACDF overwhelmingly due to its adverse events including spontaneous fusion, heterotopic ossification (HO), and relevant disc accidents.^[10–15]^ Defects existence makes the issue that which surgical procedure is optimal for the patient still controversial.

Nowadays, owing 2 operations share similar indications and surgical approaches, many spine surgeons are perplexed in surgical selection in clinical decisions. Therefore, several prospective, randomized controlled multicenter clinical trials on comparing ACDR with ACDF is performed under Food and Drug Administration (FDA) authorization.^[16–18]^ To date, the outcomes of the clinical trials indicate ACDR is superior to ACDF, and relevant meta-analysis shows consistent results. Notably, many present systematic review and meta-analysis articles tend to focus on the effectiveness,^[19–20]^ but few studies on the safety. So, which operation possess a high level of safety remains unclear.

We select primary indices including: adjacent segment degeneration (ASD), HO, Subsequent Surgical Intervention,^[21]^ gastrointestinal,^[22]^ and the secondary indices: Infection, dysphagia/dysphonia,^[23]^ neck and/or arm pain, neurological. In paper or congress, both ASD and HO remains a bone of contention stimulating scholars to think deeply in the treatment of cervical spondylosis. Subsequent Surgical Intervention could reflect the eventual outcome between 2 operations. Gastrointestinal (injury of the esophagus) complications and infection are serious adverse events that can lead to death. Dysphagia/dysphonia, neck and/or arm pain, neurological also reflex the quality of surgery. In this study, there are some key points need to be noticed. Such as different follow-ups, one or multitreated segment, and various artificial disc may influence the final outcome.

Therefore, this study aims to fill the blank and provide key evidence-based guidance for spine surgeons and designers to the evaluation of prognosis and improvement of dynamic devices. The overall adverse events comparison between ACDR and ACDF will be conducted.

### 1.1 Objective

This study aims to compare ACDR with ACDF and provide key evidence-based guidance for spine surgeons and designers to the evaluation of prognosis and improvement of dynamic devices.

## 2 Methods

### 2.1 Study design/registration

A systematic review and meta-analysis based on prospective randomized controlled trials (RCTs) was performed. This protocol was performed according to Checklist PRISMA-P (Preferred Reporting Items for Systematic review and Meta-Analysis Protocols, Supplement file 1)^[24]^ and was registered with PROSPERO 2016 (No. CRD PROSPERO, CRD42017083240, Supplement file 1). The research will be performed based on the PRISMA-P, and the Checklist PRISMA 2009 will be used to check our final results.^[25,26]^

### 2.2 Search strategy

The electric database of PubMed, Medline, Embase, Google Scholar, and Cochrane library will be systematically searched by 2 independent authors (HZ and L-JD) without region and language restriction before December 2017. The keywords will be defined as follows: anterior cervical disc replacement, cervical disc arthroplasty, cervical dynamic device, cervical artificial disc; anterior cervical decompression and fusion, anterior interbody fusion; randomized controlled trial, randomized trial, and controlled clinical trial; the keywords will be combined with Boolean operators of AND, OR, and NOT. A search strategy developed with comprehensive use of keywords is shown in Table 1. Related articles listed in previous systematic reviews, meta-analysis, and other clinical research articles will be manually searched to avoid original miss.

**Table 1:**
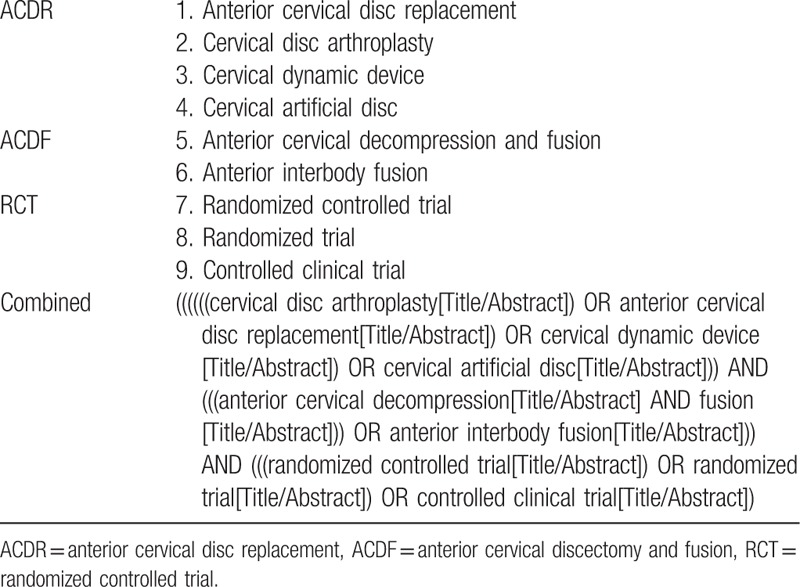
The developed search strategy for database of PubMed database.

### 2.3 Criteria of eligibility

*Inclusion*

(1) Study design: randomized controlled trials only.
(2) Participants: patients who suffered from cervical spondylosis were required surgical intervention, and must conform to strict operation indications, without limitation of age, gender, or ethnicity.
(3) Study types: research that comparing the outcomes of ACDR versus ACDF were considered.
(4) Study quality: all eligible studies must contain superior and comprehensive data.
(5) Follow-up: follow-up must be more than at a minimum of 12 months.

*Exclusion*

(1) Study design: Non-RCTs design study, case–control, case–cohort, observational studies, experimental studies, case series, and reviews will be excluded.
(2) Participants: Fail to meet operation indications for cervical spondylosis will be excluded.
(3) Study types: studies that both comparing ACDF with hybrid or cervical posterior approach techniques and comparing ACDR with hybrid or cervical posterior approach techniques will be excluded.
(4) Study quality: low quality research or with incomplete data will be excluded.
(5) Follow-up: follow-up less than 12 months follow-up will be excluded.

### 2.4 Interventions

Any anterior artificial device that was used to perform the ACDR will be included, such as ProDisc-C, Prestige disc, Bryan disc, Kineflex C, Modic-C, and PCM. The control group was treated by standard ACDF.

### 2.5 Indices measures

*Primary indices*:

(1) Adjacent segment degeneration (ASD)
(2) Heterotopic ossification (HO)
(3) Subsequent Surgical Intervention
(4) Gastrointestinal (injury of the esophagus)

*Secondary indices*:

(1) Infection
(2) Dysphagia/dysphonia
(3) Neck and/or arm pain
(4) Neurological

### 2.6 Selection process

The PRISMA 2009 flow diagram will be applied to document included and excluded studies, along with the reasons for exclusion. Two authors (YX and Z-GH) will screen the titles and abstracts independently. Duplicated, apparently irrelevant or obviously fail to meet our inclusion studies will be excluded. The rest of studies will be downloaded in full text for evaluating and inspecting the eligibility for inclusion. A study will be defined as “eligible” when both authors independently assess it as satisfying the inclusion items. When disagreement occurs, a third author will be intervened to discuss and resolve divergence.

### 2.7 Data extraction

After confirming the qualified studies for systematic review and meta-analysis, 2 authors (D-YZ and YX) will independently extract the data. A standard schedule containing basic characteristics (e.g., first author and year, study design, region, details, intervention, follow-up (months), outcomes); Primary outcomes: HO, adjacent segment degeneration (ASD), Subsequent Surgical Intervention, gastrointestinal; secondary outcomes: Infection, dysphagia/dysphonia, neck and/or arm pain, neurological. Available quantitative data will be extracted to calculate effect size. For continuous variables, the mean and standard deviation will be extracted, for dichotomous variables, the numbers of events in both ACDF and ACDR group will be extracted. Two other authors will inspect the extracted data to confirm the accuracy. All of the extracted data will be input and briefly summarized (Table 2). We will deal with missing data via contacting the originator to obtain.

**Table 2:**
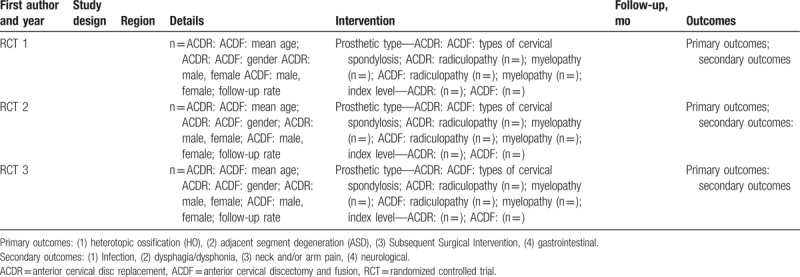
Characteristics of included studies.

### 2.8 Risk of bias assessment

The risk of bias of the included studies will be assessed according to the Furlan checklist,^[27,28]^ which includes 7 items: (A) Was the method of randomization adequate? (B) As the treatment allocation concealed? (C) Was knowledge of the allocated interventions adequately prevented during the study? (D) Were incomplete outcome data adequately addressed? (E) Are reports of the study free of suggestion of selective outcome reporting? (F) Other sources of potential bias (Table 3).

**Table 3:**
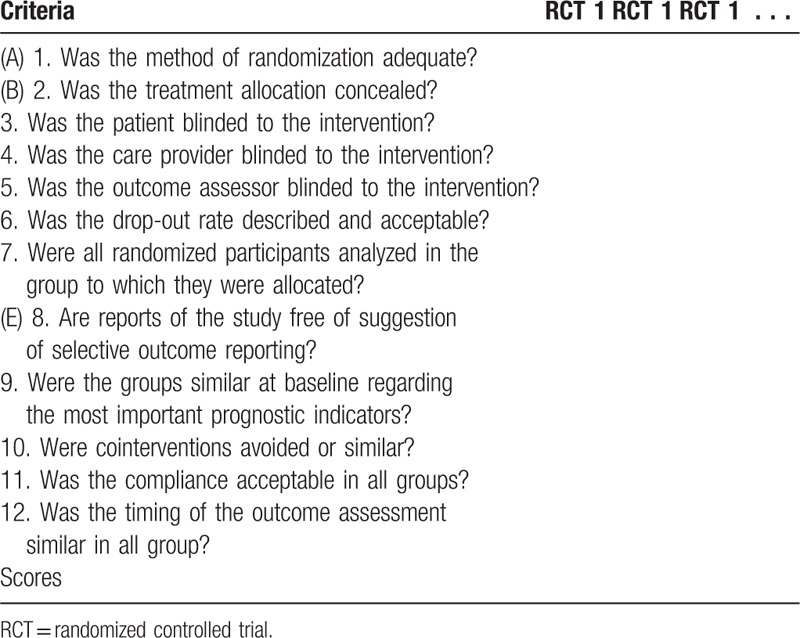
Quality assessment of included RCT studies by using the Furlan scores.

### 2.9 Data synthesis

The meta-analysis will be performed with the statistic software RevMan 5.3 software (Cochrane Collaboration, Oxford, UK). Fixed-effects models (*I*^2^ < 50%) or random-effects models (*I*^2^ ≥ 50%) will be chosen according to the heterogeneity of the included articles. For dichotomous outcomes, relative risk (RR) and 95% confidence intervals (CIs) were calculated. For continuous outcomes, weighted mean difference (WMD) and 95% confidence intervals (CIs) were calculated, insufficient data will be excluded.

### 2.10 Heterogeneity

Chi-square test and Higgin *I*^2^ test will be applied to evaluate statistical heterogeneity of included studies. A *P*-value of Chi-square test < .10 or *I*^2^ ≥ 50% indicates significant heterogeneity, however, *P*-value of Chi-square test > .10 or *I*^2^ < 50% will be acceptable.^[29]^ Subgroup meta-analysis will be performed on articles from different cervical artificial discs, patients with 1 or 2 or more pathological segments, follow-ups, and countries. Other factors such as age, gender, and race will also be conducted. Sensitivity analysis will also be conducted to examine if the included studies characteristics markedly influence the results.

### 2.11 Publication bias

Funnel plot^[30]^ will be performed by using RevMan 5.3 plug-in software to assess the publication bias, and we will use the *X* and *Y* axis as standard to distinguish obvious asymmetry that may be caused by publication bias or other factors.

### 2.12 Ethical issues and publication plan

Ethical approval is not required for the conduct of this systematic review and meta-analysis. No primary personal data will be collected, and no additional ethical approval needs to be obtained. We will disseminate the research result of this work at international or national conferences, or submit to peer-reviewed journal in spinal field. The raw data of this study will be freely available online after being accepted and published on journal.

## 3 Discussion

This systematic review and meta-analysis will, through strict methodology, identify and examine studies reporting the comparison between ACDR and ACDF. In that previous published articles are inclined to verify the effectiveness, we aim to inspect which surgical operation possesses high safety. Until now, we have searched many electronic database on website, and there is no special report on adverse event that comparing ACDR and ACDF. Therefore, we will provide evidence-based guidance for spine surgeons and designers to evaluation of prognosis and improvement of dynamic devices. Meanwhile, we will also answer the question whether the ACDR decrease ASD and increase HO comparing with ACDF at short- and long-term follow-up.

In brief, our present protocol will evaluate which surgical procedure is more safety for the treatment of cervical spondylosis. The results will be disseminated through both international conference and peer-review journal.

## Author contributions

HZ, L-JD, and XY designed the systematic review. HZ and L-JD drafted the protocol and Y-SG, Y-DY, X-ST, and D-YZ revised the manuscript. YX and Z-GH will independently screen the potential studies, extract data, assess the risk of bias, and finish data synthesis. C-HL, S-XC, and TL will arbitrate any disagreements during the review. All authors approved the publication of the protocol.

**Conceptualization:** H. Zhao, L. Duan, X. Yu.

**Data curation:** S. Chen, T. Liu, Y. Gao.

**Formal analysis:** Y. Yang.

**Investigation:** X. Tang, X. Yu.

**Methodology:** D. Zhao.

**Resources:** Y. Xiong.

**Software:** C. Li, Z. Hu.

**Supervision:** X. Yu.

**Validation:** X. Yu.

**Writing – original draft:** H. Zhao, L. Duan.

**Writing – review & editing:** H. Zhao, L. Duan.

## Supplementary Material

Supplemental Digital Content
