# Supplementary material for: Comparison of the adverse events of anterior cervical disc replacement versus anterior cervical discectomy and fusion: A protocol for a systematic review and meta-analysis of prospective randomized controlled trials
Source: Medicine (Baltimore). 2018 Apr 20;97(16):e0015. doi: 10.1097/MD.0000000000010015 (PMC5916681; doi:10.1097/MD.0000000000010015)
Supplement: Supplemental Digital Content [file medi-97-e0015-s001.pdf]

Comparison of the adverse events of anterior cervical disc replacement versus anterior cervical discectomy and fusion

Citation

Comparison of the adverse events of anterior cervical disc replacement versus anterior cervical discectomy and fusion. PROSPERO 2017 CRD42017083240 Available from: http://www.crd.york.ac.uk/PROSPERO/display_record.php?ID=CRD42017083240

Review question

1. Our current research aims to compare adverse events between artificial cervical disc replacement and anterior cervical discectomy and fusion.

2. To evaluate which surgical procedure is more safety for the treatment of cervical spondylosis.

3. Vital evidence-based guidance will be provided for spine surgeons and designers to evaluation of prognosis and improvement of dynamic devices.

Searches

The electric database of PubMed, MEDLINE, Embase, Google Scholar and The Cochrane Library will be systematically searched without language restriction in Dec. 2017 by 2 independent authors.

The keywords will be defined as follows: anterior cervical disc replacement, cervical disc arthroplasty, cervical dynamic device, cervical artificial disc; anterior cervical decompression and fusion, anterior interbody fusion; randomized controlled trial, randomized trial, and controlled clinical trial; the keywords will be combined with Boolean operators of AND, OR, and NOT.

A search strategy developed with comprehensive use of keywords is shown. Related articles listed in previous systematic reviews, meta-analysis, and other clinical research articles will be manually searched to avoid original misses.

Types of study to be included

Only randomized controlled trials with no limits on language, date or form of publication.

Condition or domain being studied

Cervical degenerative disc diseases are common in spinal disorders, and features with neck and arm pain, sometimes, associated with numbness of upper limbs, loss of function. The therapeutic methods include nonoperation, anterior cervical discectomy and fusion (ACDF), Anterior cervical disc replacement (ACDR), and others.

Participants/population

Patients who suffered from cervical spondylosis were required surgical intervention, either ACDR or ACDF.

Intervention(s), exposure(s)

Any anterior artificial device that was used to perform the ACDR will be included, such as ProDisc-C, Prestige disc, Bryan disc, KineflexlC, Modic-C, and PCM. The control group was treated by standard ACDF.

Comparator(s)/control

A control group (ACDF) is required.

Primary outcome(s)

(1) Heterotopic ossification (HO)
(2) Adjacent segment degeneration (ASD)
(3) Subsequent Surgical Intervention
(4) Gastrointestinal

Secondary outcome(s)

(1) Infection
(2) Dysphagia/dysphonia
(3) Neck /or arm pain
(4) Neurological

Data extraction (selection and coding)

Risk of bias (quality) assessment

The risk of bias of the included studies will be assessed according to the Furlan checklist, which includes 7 items: (A) Was the method of randomization adequate? (B)as the treatment allocation concealed? (C) Was knowledge of the allocated interventions adequately prevented during the study? (D) Were incomplete outcome data adequately addressed? (E) Are reports of the study free of suggestion of selective outcome reporting? (F) Other sources of potential bias. The overall quality of this systematic review and meta-analysis will be summarized and evaluated with GRADE pro (http://www.gradepro.org).

Strategy for data synthesis

The meta-analysis will be performed with the statistic software RevMan 5.3 software (CochraneCollaboration, Oxford, UK). Fixed-effects models (I^2?50%) or random-effects models (I^2?50%) will be chosen according to the heterogeneity of the included articles. For dichotomous outcomes, RR and 95% confidence intervals (CIs) were calculated, For continuous outcomes, weighted mean difference and 95% confidence intervals (CIs) were calculated.

Analysis of subgroups or subsets

Subgroup meta-analysis will be performed on articles from different countries, patients with 1or2or more pathological segments, different cervical artificial discs and different follow-ups. Other factors such as age, gender, and race will also be conducted.

Contact details for further information

He Zhao
zhaohebucm@sina.com

Organisational affiliation of the review

Department of Orthopedics III, Dongzhimen Hospital Affiliated to Beijing University of Chinese Medicine

Anticipated or actual start date

10 December 2017

Anticipated completion date

31 January 2018

Funding sources/sponsors

National 12th five-year science and technology support plan (2012BAI18B05).

Conflicts of interest

Language

English

Country
China

Stage of review
Review_Ongoing

Subject index terms status
Subject indexing assigned by CRD

Subject index terms
Diskectomy; Humans; Total Disc Replacement

Date of registration in PROSPERO
20 December 2017

Date of publication of this version
20 December 2017

Details of any existing review of the same topic by the same authors

Stage of review at time of this submission

Stage	Started	Completed
Preliminary searches	Yes	No
Piloting of the study selection process	No	No
Formal screening of search results against eligibility criteria	No	No
Data extraction	No	No
Risk of bias (quality) assessment	No	No
Data analysis	No	No

Versions
20 December 2017

PROSPERO
This information has been provided by the named contact for this review. CRD has accepted this information in good faith and registered the review in PROSPERO. CRD bears no responsibility or liability for the content of this registration record, any associated files or external websites.
